# Reconstructing the historical distribution of the Amur Leopard (*Panthera pardus orientalis*) in Northeast China based on historical records

**DOI:** 10.3897/zookeys.592.6912

**Published:** 2016-05-25

**Authors:** Li Yang, Mujiao Huang, Rui Zhang, Jiang Lv, Yueheng Ren, Zhe Jiang, Wei Zhang, Xiaofeng Luan

**Affiliations:** 1 School of Nature Conservation, Beijing Forestry University, NO.35 Tsinghua East Road Haidian District, Beijing, 100083, P. R. China

**Keywords:** Amur Leopard, Far Eastern Leopard, historical distribution, new gazetteers, Northeast China, Panthera pardus orientalis

## Abstract

The range of the Amur leopard (*Panthera pardus orientalis*) has decreased dramatically over the last 100 years. This species is still under extreme risk of extinction and conservation efforts are rigorous. Understanding the long-term dynamics of the population decline would be helpful to offer insight into the mechanism behind the decline and endangerment and improve conservation perspectives and strategies. Historical data collection has been the challenge for reconstructing the historical distribution. In China, new gazetteers having systematic compilation and considerable local ecological data can be considered as an important complementary for reconstruction. Therefore, we have set up a data set (mainly based on the new gazetteers) in order to identify the historical range of the Amur Leopard from the 1950s to 2014. The result shows that the Amur leopard was historically widely distributed with large populations in Northeastern China, but it presented a sharp decline after the 1970s. The decline appeared from the plains to the mountains and northeast to southwest since the 1950s. Long-term historical data, mainly from new gazetteers, demonstrates that such resources are capable of tracking species change through time and offers an opportunity to reduce data shortage and enhance understanding in conservation.

## Introduction

Long-term historical data would offer insight into understanding the ecological and biogeographic characteristics of population decline, and help develop predictive power for conservation management (Boakes et al. 2010; Chapron et al. 2014; Rondinini and Visconti 2015; Turvey et al. 2015). However, despite recognition of the considerable potential of long-term datasets for conservation research, policy and practice, applying long-term ecology data is still restricted by practical and conceptual barriers, including data accessibility, spatially and temporally variable and non-standardized sampling (Hortal et al. 2008; McClenachan et al. 2012; Davies et al. 2014). Therefore, recent studies always address time-scales of less than a decade (Davies et al. 2014). Only a few researchers use records over periods longer than 20 years (Vandel and Stahl 2005; Prigioni et al. 2007; Rick et al. 2012; Turvey et al. 2015). There is an increasing awareness of the need, not only to integrate historical data into conservation and environment management, but also to assess the usefulness and potential limitation of this data for developing our understanding of long-term species change.

Local gazetteers, called “*difangzhi*” (地方志), also translated as local records or annals, contain abundant information on environmental conditions and resources in China (Wen et al. 2006; Looney 2008). New gazetteers typically record considerable local ecological data from the 1950s, including animal records, as well as economic, political and demographic information (Looney 2008; Xue 2010). Most of them are compiled following a specific “scientific” natural history tradition and this makes it easy to identify the species records. In addition, compilation of gazetteers is systematized and provides a geographical coverage across most of China. For the records from field surveys, museums and papers, new gazetteers may have been neglected for a long time. This may be seen as reasonable, because new gazetteers are only concerned with species which are economic, unique or common. Recent research, however, highlights that this resource can provide effective records for reconstructing long-term population dynamics (Turvey et al. 2015; Zhang et al. 2016). In this case, we suggest that new gazetteers can be an irreplaceable source for conservation biology resources.

The Amur leopard, or Far Eastern leopard (*Panthera pardus orientalis*) is considered to be one of the most endangered subspecies in the world and has been listed as critically endangered on the IUCN red list since 1996 (Uphyrkina et al. 2002). Compared to the distribution in the late 19th century, the range of the Amur leopard has decreased dramatically over the last 100 years. Recent research shows that there are only 14–20 adults and 5–6 cubs in the southwestern Primorye region of Russia (Henschel et al. 2008; Spitzen et al. 2012); Perhaps some may still occur in North Korea, although their status there is uncertain (Uphyrkina et al. 2002); Fewer than 10 leopards likely existed in Jilin and Heilongjiang Provinces of northeast China in the later 1990s, but the population seems to have increased in recent years (42 leopards were photographed, including 40 adults (21 males, 17 females, and two of unknown sex) and two cubs) (Yang et al. 1999; Wang et al. 2015). The tiny population that survives today is under extreme risk of extinction because of poaching, deforestation, inbreeding, and anthropogenic pressure (Ma 1989; Yang et al. 1999; Yang et al. 2000; Piao et al. 2011; Hebblewhite et al. 2011; Kelly et al. 2013; Cat Specialist Group 2014). Therefore, a basic understanding of their distribution over decades is necessary to design conservation strategies on a regional scale. To address this problem, we suggest that new gazetteers can be a useful resource. Combining new gazetteers, which regularly have Amur leopard records, with historical information from literature, news and scientific surveys, we identified the range of the Amur leopard in Northeast China from the 1950s to the 1990s. The historical dynamic for the Amur leopard in Northeast China was then reconstructed primitively by integrating historical records within a geographic information system (GIS).

## Materials and methods

### Area

The study area comprises Heilongjiang province, Jilin province and northeast of Inner Mongolia covered by temperate forest and boreal forest (N40°5'~53°17', E115°30'~135°06', about 938000 km^2^ with a forested area of 402,000 km^2^), which is the most important forestry and agricultural production base in China. The climate is a continental monsoon climate with a negative water balance. The annual precipitation is 400–1000mm, and the annual average temperature is 1–4°C, with the north - south temperature gradient of 25. The region includes coniferous forest, broad-leaved mixed forest, secondary forest, woodland shrub and marshy grass areas, and contains more than 2500 plant species (Zhou 1997; Hou 2001). Coniferous forest is mainly located in the Greater Khingan Mountains where it is characterized by *Larix gmelinii*. The area of broad-leaved mixed forest includes the Lesser Khingan Mountains, Changbai Mountains and Wanda Mountains dominated by *Larix gmelinii*, *Pinus koraiensis* and *Betula platyphylla* (Cheng & Yan, 2008). Forest ecosystems in Northeast China support a number of forest-dependent carnivores and herbivores, for instance the Amur leopard and its potential prey – the Siberian roe deer (*Capreolus pygargus*), wild boar (*Sus scrofa*) and sika deer (*Cervus nippon*).

### Data

Amur leopard historical records were obtained in the following five ways:

(1) New gazetteers. Due to being an important cultural symbol, economic species and having an ecological niche in human history and culture, large carnivores like the Amur leopard are recorded regularly in new gazetteers. The data from new gazetteers can be considered as the hard fact, because they came from the fur trade records, hunter records, sightings, wild life surveys and conflicts. Systematic compilation made it possible to divide the records into different periods and to cover most of China. Also utilizing fauna and nature reserve scientific surveys, papers, scientific research and news, it is possible to compile substantial records for a long period and covering a large geographical area. In this paper, more than 90% of historical records came from new gazetteers. They were obtained from the National Library of China (http://www.nlc.gov.cn/), Duxiu Search Engine (http://www.duxiu.com/), and the Wanfang database (http://www.wanfangdata.com.cn/). Relevant information was found with the keywords – “Amur leopard”, “Far Eastern Leopard” and “*Panthera pardus orientalis*”.

(2) Fauna surveys. Fauna surveys recorded the specimen data with geographic information. We collected the distribution data from 9 Fauna surveys (Shou. 1962; Ma et al. 1986; Gao et al. 1987; Ma et al. 1989; Wu. 1993; Wang. 1998; Wang et al. 1998; Sheng et al. 1999; Zhao et al. 1999; State Forestry Administration of the People’s Republic of China 2009).

(3) Nature reserve scientific surveys. The relevant data was collected from the National Library of China and the School of Nature Conservation, Beijing Forestry University.

(4) Papers and scientific research. The keyword “Amur leopard”, “Far Eastern Leopard” and “*Panthera pardus orientalis*” were used to search several online databases such as the China National Knowledge Internet, Wanfang Database, Duxiu Search Engine, Google scholar (https://scholar.google.com/) and Biodiversity Heritage Library (http://www.biodiversitylibrary.org/). Records from 1998 mainly came from surveys (WCS program) in Heilongjiang and Jilin provinces.

(5) News. The keywords “Amur leopard”, “Far Eastern Leopard” and “*Panthera pardus orientalis*” were used to search several online databases such as the China National Knowledge Internet, Wanfang Database, Duxiu Search Engine, Science Daily and Google. Records with detail location associated with photograph or video were effective.

### Data analysis

Filters are essential for further analysis, because historical records may contain potential errors or uncertainties. Records from four of the ways above (2-5) were integrated into a dataset with brief information on the area of occupancy, and then this is compared with the gazetteer records. Records that conflicted with the dataset without any hard facts, including relevant or detailed descriptions, were excluded from analysis. All Chinese-language records were translated directly by the authors.

Maps of Northeast China (1:1,000,000) were acquired from the National Geographic Information Bureau, and spatial coordinates of all occurrence records were assigned using Google Earth. Multiple occurrences at matching sites, such as repetitions of the same record or when the distance between locations was less than 5km were excluded. Records from killed animals, attacks on humans, prey remains, claw traces, footprints and photographs were excluded if they were repeated. Valid records were assigned with coordinates from public resources such as Google Earth v 7.1.2, classified into different decades (1950s, 1960s, 1970s, 1980s, 1990s and 2000–2014).

Amur leopard occurrence positions were then uploaded into ArcGIS 10.2, and overlaid with layers representing altitude (http://srtm.csi.cgiar.org/) and the borders of administrative regions. Using the number and intensity of records, it is possible to reconstruct and evaluate the distribution change in different decades of the Amur leopard population.

## Results

A total of 84 documents of the 2235 new gazetteers examined presented information on the Amur leopard. These provided evidence regarding 294 Amur leopard records, while another 169 Amur leopard records were retrieved from the other four sources (as mentioned in the data sources section (2)-(5), giving a total of 463 Amur leopard records). Some records were excluded (as mentioned in the methods section) and finally 341 Amur leopard records were mapped and used for further analysis (Suppl. material S1).

New gazetteers were considered as the main resource before the 1990s. In the 1950s, 96.4% of total records came from new gazetteers. 36.2% of the new gazetteers records were confirmed by fauna and papers. 97.5%, 96.3% and 93.9% of total records came from new gazetteers in the 1960s, 1970s and 1980s respectively. 42.4%, 41.2%, and 54.0% of the new gazetteers records were confirmed by fauna, papers and nature reserve scientific surveys in the 1960s, 1970s and 1980s respectively. New gazetteers were still an important resource in the 1990s (44.7% of total in the 1990s) (Suppl. material S1). Scientific research for Amur leopard provided 17 records. Nature reserve scientific surveys and news dominated after 2000 and only one record came from new gazetteers.

**Figure 1.**
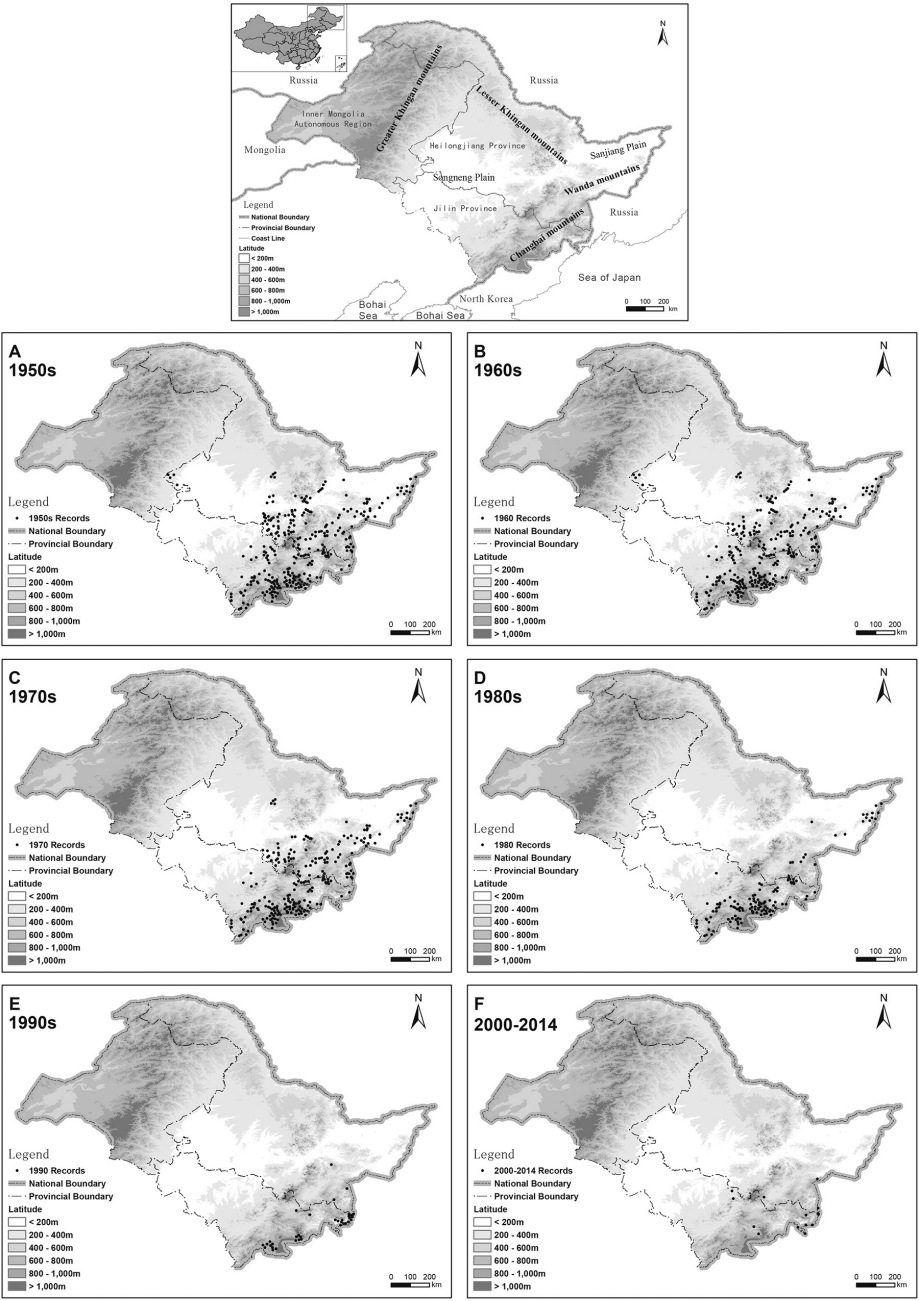
Amur leopard distribution in different periods.

Our result implied that the Amur leopard was widely distributed with a large population in Northeast China - Southern Lesser Khingan Mountains, Changbai Mountain and Wanda mountains (Fig. 1). Before 1970, the Amur leopard occurrence points didn’t present obvious alterations (Fig. 1A, B). In the 1970s, the situation deteriorated continually. Compared with the distribution in the 1950s, there was a reduction of 19.6% of records in the 1970s. From the 1980s onwards the Amur leopard became limited to the core area of the Wanda Mountains and Changbai Mountains. There was a decline of 56.1% of records in the 1980s (Fig. 1C, D). In the 1990s, there were only a few records limited to several places, for example Hunchun, Wanqing, Yanji and Helong. Afterwards, the distribution became concentrated in the Hunchun and Wanqing in 2000–2014 (Fig. 1E, F). In addition, records in areas higher than 200m continuously increased before 2000, but a slight decrease presented in 2000–2014 (1950s: 79%; 1960s: 81%; 1970s: 84%; 1980s: 89%; 1990s: 92%; 2000–2014: 88%).

## Discussion

Our investigation of the potential of using long-term historical records to reconstruct long-term population dynamics shows that new gazetteers along with multiple effective resources can contribute novel insights for tracking the target species dynamics across longer timescales than is usually addressed in ecology or conservation biology. However, it is inevitable that the new gazetteer data compiled by non-scientific observers cannot provide a complete species records at the standard typically expected by modern ecologists, even if combined with multiple resources (2-5 resources). For example, hunt archives are the major component in new gazetteer records (like “Amur leopard was killed by snare in Liushuihe Forest Farm in 1964, winter”). Since hunting was banned in the 1980s, such records decreased sharply and can increase omission error after 1990. Spatial and temporal bias in new gazetteer records also generates deviation. For this reason, our investigation highlights some ways to reduce the commission or omission errors in new gazetteer records, including choosing identifiable and economic species, no generic species absent in the same area, and integrating with scientific data. Indeed, it is possible that the Amur leopard still exists in an area or specific period with no record, or be present in contrary circumstances. However, such historical dynamics for the Amur leopard in Northeast China still reveals some aspects of the pattern and process of population decline across more than 50 years that cannot be fully understood by the present-day population: The Amur leopard population presents a sharp decline after the 1970s; and the decline is likely to appear from the plain to the mountain and northeast to southwest, which implies negative impact from anthropogenic pressure.

Some questions still need to be clarified. For example, the Amur leopard population in the Greater Khingan Mountains is elusive (the range is unknown, but they are present). Only one fauna researcher (Gao et al. 1987) provides one record in Horqin Right Front Banner, Inner Mongolia autonomous region in the southwestern Greater Khingan Mountains. Four other fauna researchers describe the distribution briefly (Ma et al. 1986, 1989; Wu 1993; Zhao et al. 1999), and two of four suggest that local population have been extinct in the 1970s with none appearing in hunting or witness records after 1970 (Ma et al. 1989; Wu 1993). An extract taken from the Longjiang xianzhi says” the leopard was bountiful at the time of the foundation of the PR of China”. Longjiang town is about 80km away from the north of the Horqin Right Front Banner. It is safe to say that the Amur leopard was distributed in the Greater Khingan Mountains in the 1950s, but the range change is still unclear. Moreover, higher latitude records increased before 2000 and this implies that the negative impacts under anthropogenic pressure for the Amur leopard and the decline in 2000-2014 can be either a promising sign on the effectiveness of the conservation strategy, or such a phenomenon may just be associated with data shortage.

Our reconstruction of the dynamics of the Amur leopard population decline enlightens a new case study with new perspectives shows that the historical records have considerable potential to contribute to ecological baselines for informing conservationists. We recommend further investigation to not only detect feasibility for reconstructing the dynamics of other species in China by Chinese gazetteers, or quantifying the human pressure by the response of wildlife over time in modern China, but also to evaluate the possibility of revealing the potential distribution in the past with the historical records and distribution models. Furthermore, we encourage conservation researchers and practitioners to consider this data type (not only the gazetteers, but also other data resource considered as local historical record around the world) as complementary, in order to reduce data shortage and enhance understanding in conservation. Sufficient data would provide greater opportunities for conservationists and environmental planners to put plans in place that would reduce the danger of endangered species going extinct in China and elsewhere.

## Supplementary materials

Supplementary material 1 Distribution information from new gazetteers records and from other resources Data type: PDF file This dataset is made available under the Open Database License (http://opendatacommons.org/licenses/odbl/1.0/). The Open Database License (ODbL) is a license agreement intended to allow users to freely share, modify, and use this Dataset while maintaining this same freedom for others, provided that the original source and author(s) are credited.Li Yang, Mujiao Huang, Rui Zhang, Jiang Lv, Yueheng Ren, Zhe Jiang, Wei Zhang, Xiaofeng Luan

Supplementary material 2 Records in different periods from new gazetteers records Data type: PDF file This dataset is made available under the Open Database License (http://opendatacommons.org/licenses/odbl/1.0/). The Open Database License (ODbL) is a license agreement intended to allow users to freely share, modify, and use this Dataset while maintaining this same freedom for others, provided that the original source and author(s) are credited.Li Yang, Mujiao Huang, Rui Zhang, Jiang Lv, Yueheng Ren, Zhe Jiang, Wei Zhang, Xiaofeng Luan
