# Supplementary material for: Reconstructing the historical distribution of the Amur Leopard (Panthera pardus orientalis) in Northeast China based on historical records
Source: Zookeys. 2016 May 25;(592):143–53. doi: 10.3897/zookeys.592.6912 (PMC4926640; doi:10.3897/zookeys.592.6912)
Supplement: Supplementary material 1 — Distribution information from new gazetteers records and from other resources [file zookeys-592-143-s001.pdf]

Appendix S1. Distribution information from new gazetteers records

Province	*Title*	*Last record period*	*Records*
Heilongjiang	Acheng Xianzhi	1950s	Bao (Amur leopard)
Heilongjiang	Bamiantong LinyejuZhi	1960s	Bao (Amur leopard) was distributed in Fengyueqiao and Maqiaohe.<br>An Amur leopard was killed by truck in Fengyuanqiao in 1965
Heilongjiang	Dahailin LinyejuZhi	1950s	Jinqian bao (Amur leopard)<br>An Amur leopard was captured in NO. 151 sawmill in 1953 by Chui Yunfeng.<br>An Amur leopard was killed in Lishugou in 1956<br>An Amur leopard was killed in Liushuhe Forestry Farm in 1964 in winter
Heilongjiang	Dongning XianZhi	1970s	Bao (Amur leopard)<br>A woman was killed by Amur leopard on the road from Xishan to Huluobowei in autumn in 1978
Heilongjiang	HaiLin XianZhi	1970s	Jinqian bao (Amur leopard)
Heilongjiang	Heilongjiang ShengZhi	1980s	Jinqianbao (Amur leopard) was distributed in Qixinglizi Nature Reserve
Heilongjiang	Linkou LinyejuZhi	1970s	Jinqian bao (Amur leopard)
Heilongjiang	Longjiang Environmental Protection Zhi	1960s	Bao (Amur leopard)
Heilongjiang	Mudanjiang ShiZhi	1970s	Bao (Amur leopard)
Heilongjiang	Qitaihe Shi LinyeZhi	1970s	Tubao (Amur leopard)
Heilongjiang	Shangzhi XianZhi	1970s	Jinqian bao (Amur leopard) was distributed in the area from Zhangguangcailing mountain to DaTuDingZi mountain
Heilongjiang	Shuangyashan ShiZhi	1970s	Bao (Amur leopard)
Heilongjiang	Suifenhe ShiZhi	1980s	Jinqian bao (Amur leopard)
Heilongjiang	Suiyang LinyejuZhi	1970s	Bao (Amur leopard)
Heilongjiang	Tangyuan XianZhi	1960s	Bao (Amur leopard)
Heilongjiang	Wuchang LinyeZhi	1970s	Bao (Amur leopard)
Heilongjiang	Yanshou XianZhi	1950s	Bao (Amur leopard)
Heilongjiang	Yunshan Nongchangzhi	1980s	Bao (Amur leopard)
Helongjiang	Baoqing Xianzhi	1970s	Jinqian bao (Amur leopard)
Helongjiang	Bawuer Nongchangzhi	1950s	Jinqian bao (Amur leopard)<br>An witness in 1956
Helongjiang	Bayan XianZhi	1950s	Bao (Amur leopard) was disappeared since 1950s
Helongjiang	Bin XianZhi	1950s	Jinqian bao (Amur leopard)
Helongjiang	Dongfanghong LinyejuZhi	1980s	Bao (Amur leopard) was distributed in Qiyuan, Wulingdong, Yongxing, Shichang and Dayake
Helongjiang	Fangzheng XianZhi	1970s	Jinqian bao (Amur leopard)
Helongjiang	Jidong XianZhi	1970s	Baozi (Amur leopard)
Helongjiang	Linkou XianZhi	1970s	Jinqian bao (Amur leopard)

Helongjiang	Mishan XianZhi	1970s	Bao (Amur leopard)
Helongjiang	Mudangjiang Guanlijuzhi	1980s	Bao (Amur leopard) was distributed in Mudanjiangshi, Mishanxian, Hailinxian, Ninganxian and Jidongxian
Helongjiang	Mudanjiang Difanglinyezhi	2005	Jinqian Bao (Amur leopard)<br>An witness in Xiaobeihu Forestry Farm in Ningan in 2005
Helongjiang	Mudanjiangshi Huanjingbaohuzhi	1980s	Dongbei Bao (Amur leopard)<br>47 individual in Mudanjiangshi from local research in 1980
Helongjiang	Ningan XianZhi	1970s	Bao (Amur leopard)
Helongjiang	Raohe XianZhi	1980s	Jinqian bao (Amur leopard)
Helongjiang	Songhuajiang DiquZhi	1970s	Jinqian bao (Amur leopard) was distributed in Heilonggong in Shangzhi and Yuejin Forestry Farm in Yabuli
Helongjiang	Suiling XianZhi	1970s	Bao (Amur leopard)
Helongjiang	Tonghe XianZhi	1960s	Bao (Amur leopard)
Helongjiang	Weihe LinyejuZhi	1970s	Bao (Amur leopard)
Helongjiang	Yichun Shizhi	1980s	Bao (Amur leopard) was distributed in southern Yichun
Jilin	Antu Xianzhi	1980s	Bao (Amur leopard) distributed in Southern Huanggouling
Jilin	Bajiazi LinyejuZhi	1980s	Jinqian bao (Amur leopard)
Jilin	Dunhuaxian Wildlife JianZhi	1980s	Jinqian bao (Amur leopard) distributed in Dapuchai, Linsheng and Dashitou
Jilin	Fusong XianZhi	1970s	Jinqian bao (Amur leopard)
Jilin	Helong LinyejuZhi	1990s	Tubaozi (Amur leopard)
Jilin	Helong ShiZhi	1990s	Jinqian bao (Amur leopard)
Jilin	Helong Xian LinyeZhi	1980s	Jinqian bao (Amur leopard)
Jilin	Hongshili ZhenZhi	1980s	Bao (Amur leopard)
Jilin	Huadian XianLinyeZhi	1980s	Bao (Amur leopard)
Jilin	Huinan Forestry Management Ju Zhi	1980s	Jinqian bao (Amur leopard)
Jilin	Hunchun ShiZhi	1990s	Jinqian bao (Amur leopard)
Jilin	Hunjiang XianZhi	1980s	Jinqian bao (Amur leopard)
Jilin	Jian XianZhi	1980s	Bao (Amur leopard)
Jilin	Jiaohe XianZhi	1960s	Jinqian bao (Amur leopard)
Jilin	Jingyu XianZhi	1970s	Bao (Amur leopard)
Jilin	Linjiang LinyejuZhi	1990s	Jinqian bao (Amur leopard)
Jilin	Longjing XianZhi	1980s	Tubaozi (Amur leopard)
Jilin	Quanyang LinyejuZhi	1980s	Jinqian bao (Amur leopard)
Jilin	Taishang ZhenZhi	1980s	Bao (Amur leopard)
Jilin	Tianqiaoling LinyejuZhi	1980s	Jinqian bao (Amur leopard)
Jilin	Tonghua ShiZhi	1980s	Jinqian bao (Amur leopard)
Jilin	Tonghua XianZhi	1980s	Bao (Amur leopard)

Jilin	Wulajie XiangzhenZhi	1980s	Tubao (Amur leopard)
Jilin	Yanji ShiZhi	1980s	Bao (Amur leopard)
Jilin	YongZhi ShiZhi	1970s	Bao (Amur leopard)
Jilin	YongZhi XianZhi	1970s	Bao (Amur leopard)

Appendix S2. Important distribution information from other resources

Resource	*Type*	*Year*	*Location*
Article	Footprint	2004	Madida Reserve station of Hunchun Nature Reserve
Article	Footprint and witness	2004	Qinglongtai Reserve station of Hunchun Nature Reserve
Article	Infrared camera	2004	Hunchun Nature Reserve
Article	Interview	1970	Two Amur leopard were shoot in Erdao Town
Fauna (Beast investigation report in Northeast)	specimen	1950s	Baoqing (Heilongjiang Province), Wangqing, Antu(Jilin Province)
Fauna (China Fauna:Beast Volume)	specimen	1980s	Amur leopard was distributed in Baoqing, Mudanjiang, Dongning, Muling (Heilongjiang); Wangqing, Hunchun, Helong, Dunhua, Yanji, Antu, Changbai, Jian, Fusong, Tonghua, Jingyu, Hunjiang, Liuhe, Huinan, Jiaohe, Shulan, Huadian (Jilin); Keyouqianqi (Inner Mongolia Autonomous Region)
Fauna (Wild animal of the southwest slope of Changbai Mountain)	specimen	1980s	Bao (Amur leopard) distributed in Shiyidaogou, Shisandaogou, Malugou, Baoquanshan, Fusong manjiang in Changbai and Dongbeicha in Hunjiang
Hunchun Nature Reserve	specimen	1980s	Amur leopard was killed in Yangpao Xiang
Scientific investigation	investigation	1992	2 indiv. in Madida, Qiuligou and Chunhua, another 1-2 indiv. in Duhuangzi and Xinancha
Scientific investigation in Jilin in 1998	Footprints	1997	Footprints in Huangbaishugou
	Food debris	1996	Food debris in Xibeichagou
	Witness	1995 and 1998	Two Amur leopard were tracking the deers in Yingzuilazi. Another witness is in Guancailazi in 1995
		1996-1997	Witness in Caomaocun in Chunhua
		1997-1998	Witness in Qingniwa Donggou and

			Tianjiazhai
		1997	Witness in Guchengshan, Lanjiatangzicun in Chunhua
		1996	Witness in Dahuanggou Forestry Farm and Sandaogou Forestry Farm
		1998	Witness in Yushuxiang
		1998	Footprints in Qingniwa Donggou
Scientific investigation in Heilongjiang in 1998	Witness	1996 and 1998	Witness in Shuangqiaozi Forestry Farm
		1997-1998	Witness in Laoheishan Forestry Farm
		1997-1998	Witness in Gonghe Forestry Farm
News	Infrared camera and footprint	2011	Amur leopard was record in Xinancha Forestry Farm
News	attack	1999	Two man were attacked in Naozhigou in Hadamen Xiang
News	Food debris	2014	Badao town of Shulan city,Jilin Province
News	Food debris	2013	Jinsong Forestry of Baihe Forestry Bureau
News	Footprint	2011	Duhuangzi Forestry and Xinancha Forestry of Wangqing Forestry Bureau
News	Footprint and food debris	2014	Zhengyang town of Shulan city,Jilin Province
News	Footprints	2012	Dongqing Forestry of Antu Forestry Bureau
News	Infrared camera and footprint	2014	Chaoyanggou Forestry
News	Photos and videos	2015	Mengling town of Hunchun city,Jilin Province
News	Video Record	2011	Jingxin town of Hunchun city, Jilin Province
News	Video Record	2014	Wujia mountain in Jingxin town, Jilin Province
