# Supplementary material for: Reconstructing the historical distribution of the Amur Leopard (Panthera pardus orientalis) in Northeast China based on historical records
Source: Zookeys. 2016 May 25;(592):143–53. doi: 10.3897/zookeys.592.6912 (PMC4926640; doi:10.3897/zookeys.592.6912)
Supplement: Supplementary material 2 — Records in different periods from new gazetteers records [file zookeys-592-143-s002.pdf]

Table S1. Records in different periods

Resource	*1950s*	*1960s*	*1970s*	*1980s*	*1990s*	*2000-2014*
Fauna	114	114	101	74		
Papers	8	8	4			
Nature reserve scientific surveys				2	1	2
New gazetteers	294	269	232	124	17	
Kill records	2*	2*	2	1		
Attack human records			1*		1	
Prey remains and claw traces					3	4
Excreta					1	
Footprints					7	7
Photographs						6
Witness	2*				10	2(1*)
Exclude records (repeat records)	111	115	98	69	1	4
Total	305	278	242	132	38	17

"" means that such records came from new gazetteers; one witness record in the 2000s came from new gazetteers*
